# Age-aware constitutive materials model for a 3D printed polymeric foam

**DOI:** 10.1038/s41598-019-52298-z

**Published:** 2019-11-04

**Authors:** A. Maiti, W. Small, J. P. Lewicki, S. C. Chinn, T. S. Wilson, A. P. Saab

**Affiliations:** Lawrence Livermore National Laboratory, Livermore, CA 94550 USA

**Keywords:** Mechanical properties, Polymers

## Abstract

Traditional open or closed-cell stochastic elastomeric foams have wide-ranging applications in numerous industries: from thermal insulation, shock absorbing/gap-filling support cushions, packaging, to light-weight structural and positional components. Recent developments in 3D printing technologies by direct ink-write have opened the possibility of replacing stochastic foam parts by more controlled printed micro-structures with superior stress-distribution and longer functional life. For successful deployment as mechanical support or structural components, it is crucial to characterize the response of such printed materials to long-term external loads in terms of stress-strain behavior evolution and in terms of irreversible structural and load-bearing capacity changes over time. To this end, here we report a thermal-age-aware constitutive model for a 3D printed close-packed foam structure under compression. The model is based on the Ogden hyperfoam strain-energy functional within the framework of Tobolsky two-network scheme. It accurately describes experimentally measured stress-strain response, compression set, and load retention for various aging times and temperatures. Through the technique of time-temperature-superposition the model enables the prediction of long-term changes along with the quantification of uncertainty stemming from sample-to-sample variation and measurement noise. All aging parameters appear to possess the same Arrhenius activation barrier, which suggests a single dominant aging mechanism at the molecular/network level.

## Introduction

3D printing, also known as Additive Manufacturing (AM), has revolutionized a diverse range of application areas and industries^1^ including, biological applications such as tissue and organ engineering, medical devices, and drug delivery. In the automotive, aerospace, construction, and packaging industries AM applications now include robust structural components, *in situ* sensor development, and material support for intricate microstructural design and integration. A relatively novel mechanical application involves 3D printed polymeric *foams*^2^, which are uniform layered structures with voids or spaced gaps. Such materials, whose architecture and porosity can be easily tailored, are showing great promise as attractive alternatives for traditional cellular materials^3–5^ like stochastic foams and corks as impact-absorbing support cushions that also provide striction for mechanical stability. For many such applications, cushions are under constant compressive stress for extended time periods (year to decades), during which these components may be exposed to diverse temperatures, humidity, radiation, and chemical environments that can lead to permanent chemical/structural and irreversible mechanical behavior changes to the material^6,7^.

In a previous contribution the authors demonstrated that while under compression, 3D printed polymeric “foams” display improved long-term structural stability as compared to their stochastic counterparts^8^. This, despite the bulk polymeric resin of the 3D printed material being more prone to irreversible deformation relative to the constituent resin of the stochastic foam, necessitated by manufacturing constraints. The enhanced stability of the AM foam was attributed to superior stress distribution within its more uniform architecture. Two metrics, each dependent on time (*t*) and temperature (*T*), were used to quantify the long-term stability of the foam, i.e., (1) compression set (*CS*), defined as the irreversible loss in foam thickness as a ratio of the original compression level; and (2) load retention (*LR*), defined as the load (force) necessary to compress the foam to the original level as a ratio of the load needed at time *t* = 0.

Physically, non-zero *CS* represents a permanent structural deformation (e.g., the formation of gaps) in an aged cushion upon the release of long-term stress, while the decrease of *LR* from 100% represents a loss in the cushion’s weight-supporting strength. Both these quantities have been used in the literature to characterize the aging of elastomeric and polymeric foam materials^7,9^. In the absence of measurement noise, *CS* monotonically increases and *LR* monotonically decreases with time, with the rates of both such changes being accelerated at higher temperatures. However, the quantities *CS* and *LR* are not independent but are tied together by changes in the underlying polymer network structure of the foam that also govern the stress-strain response characteristics at the macroscopic level.

The goal of the work reported here was to create a simple materials model that can quantitatively describe the evolution of *CS* and *LR* on a unified footing. With an eye to performing future finite-elements simulations at the parts level, we wanted the materials model to be constitutive, i.e., one in which the foam is treated as a continuum. The model is based on the following physical picture: initially (*t* = 0) there is a single network that governs the mechanical response of the foam. Under long-term strain (compression in our case) a second network forms that is in equilibrium at the strained state. While the material is under strain, the first network progressively weakens (i.e., decreases in modulus), while the second network becomes stronger, with a resulting shift in the equilibrium thickness that results in *CS*. On the other hand, *LR* results from a joint effect of the altered equilibrium thickness and changes in mechanical modulus in both networks.

## AM FCT Foam

**Figure 1:**
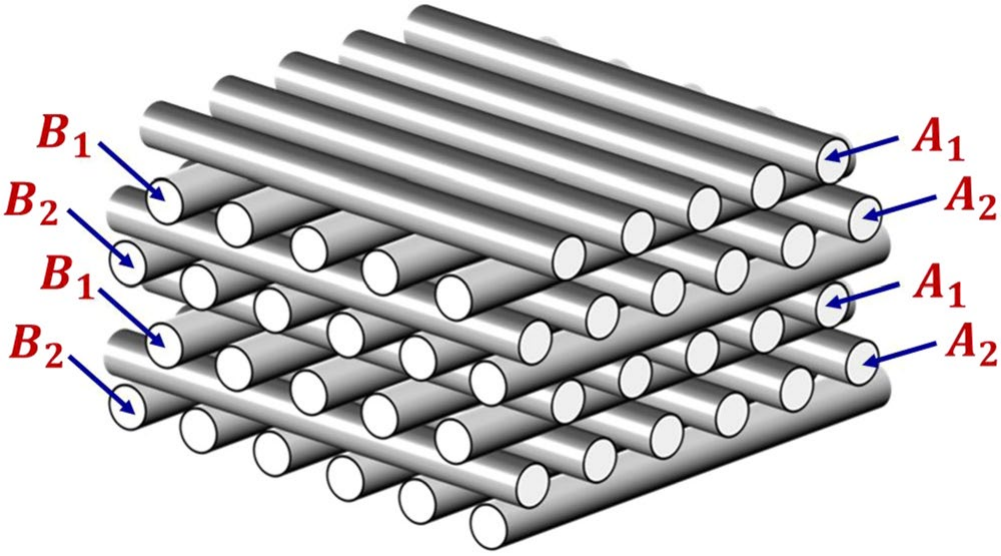
Schematic diagram of a 3D printed foam with the face-centered tetragonal (FCT) architecture used in the present study. It is made of Dow Corning’s polysiloxane-based silica-filled elastomer, called SE-1700. Each cylindrical strand is of diameter 250 μm with a small overlap between perpendicular cylinders at each junction, resulting in a total thickness of ~1.6 mm for the eight-layer structure. Initially printed samples had in-plane dimensions 75 × 75 mm square (uniform thickness 1.6 mm), out of which circular discs of diameter 28.7 mm were cut for use with aging and mechanical response study.

The foam specimen used in this work was additively manufactured using the method described previously^8^. Briefly, to print the AM foam, a commercially available silica-reinforced PDMS elastomer (Dow Corning SE 1700 adhesive) was used. A 1.6 mm thick eight-layer structure was produced with 250-μm-diameter strands, spaced to yield ~47% porosity. Based on the manufacturer’s recommendation the AM FCT foam was cured at 150 °C for 1 hour. The foam used in the present study was printed with a face-centered tetragonal (FCT) architecture defined by repeated layer-arrangement (*A*_1_*B*_1_*A*_2_*B*_2_)_*n*_ where the strands in layers *A*, *B* are mutually perpendicular to each other, and the strands in the layers *A*_1_, *A*_2_ (and also in layers *B*_1_, *B*_2_) are shifted with respect to each other by half-a-pitch (see Fig. 1). As previous work has demonstrated, such architectures have favorable stress distribution properties and do not suffer from stress-concentration induced structural instabilities associated with non-close-packed, e.g., simple-cubic structures^2^. Initially printed samples were of lateral dimensions 75 × 75 mm square (uniform thickness 1.6 mm), out of which circular discs of diameter 28.7 mm were cut for use with aging and mechanical response studies as described below.

## Experimental Measurements of Stress-Strain Response

Specimens were compressed in rigs comprised of two parallel steel plates bolted together with a given separation to achieve an aging compressive strain of 42%, almost 90% of lock-up. For a schematic diagram of the apparatus, see ref.^8^ and its supplemental info. Compressed specimens were aged at four different temperatures (room temperature (22.5 °C), 30 °C, 50 °C, and 70 °C) under a nitrogen environment for a total period of 14 months. At specific time points (24 h, 1 week, 2 weeks, 4 weeks, and monthly thereafter) the uncompressed specimen thickness and load at the aging strain were measured using an Instron 5500R dual-column load frame. Heated specimens were allowed to cool under compression for 24 h prior to measurement in an air atmosphere. The compression rig containing the specimen was positioned in the Instron (air atmosphere) and the bolts were removed while under compressive load. Three unload-load cycles were performed (test speed = 0.2 mm/min), and the load and crosshead displacement were recorded at 10 Hz. The bolts were put back in place and aging of the compressed specimen under nitrogen was resumed. Instrument compliance (~10^−4^ mm/N) was determined using an empty compression rig to correct for the crosshead displacement. The recovered thickness of the uncompressed specimen was determined based on the compliance-corrected displacement of the crosshead when the load first began to increase during the third loading phase. Finite strain rates in our measurements and the presence of hysteresis led to small differences in stress response between unloading and loading and also between the different cycles, with the differences becoming the smallest in the third cycle. A full modeling of such phenomenon would require the analysis of stress relaxation through viscoelastic terms^10,11^. To avoid such complexity, in all cases below, we model the loading curve of the last (i.e., third) cycle.

## Ogden Hyperfoam + Two-network Tobolsky

One of the most successful models to describe the stress-strain response of traditional cellular foams was originally formulated by Ogden^12^ and subsequently refined by Hill^13^ and Storakers^14^. Many other hyperelastic models, based on both strain invariants and eigenvalues of deformation gradient tensor have been developed over the years^15^, especially for incompressible elastomers. And many such models can be extended to foam systems through the addition of a volume-changing term^16^. However, the Ogden-Hill-Storakers model, commonly known as the Ogden hyperfoam model, is considered the most flexible, both for modeling static as well as time-dependent response^11,17^ (through the addition of viscoelastic terms). The Ogden hyperfoam model involves a strain energy functional *ψ* that is expressed as a series of *N* terms, each term of the series having three parameters, i.e., *μ*, *α*, *β*, where *μ* is related to the shear modulus, *β* is related to the Poisson’s ratio, and *α* is an exponent (which can be either positive or negative) as seen in Eq. (1) below:

1 $${\psi }_{Hyp}({\lambda }_{1},{\lambda }_{2},{\lambda }_{3})=\mathop{\sum }\limits_{k=1}^{N}\,\frac{2{\mu }_{k}}{{\alpha }_{k}^{2}}[{\lambda }_{1}^{{\alpha }_{k}}+{\lambda }_{2}^{{\alpha }_{k}}+{\lambda }_{3}^{{\alpha }_{k}}-3+\frac{1}{{\beta }_{k}}\{{({\lambda }_{1}{\lambda }_{2}{\lambda }_{3})}^{-{\alpha }_{k}{\beta }_{k}}-1\}].$$

Here *λ*_1_, *λ*_2_, *λ*_3_ are the three principal stretch ratios, which are obtained as the square-root of the eigenvalues of the right Cauchy-Green tensor^16^. The principal Piola-Kirchhoff stresses (*P*_*a*_) (also called the engineering stress) are obtained from the strain energy function by the formula:

2 $${P}_{a}=\frac{\partial {\psi }_{Hyp}}{\partial {\lambda }_{a}},\,a=1,2,3$$

In the case of uniaxial compression (with compression being in direction 1) we have *λ*_1_ = 1 − *ε*, *λ*_2_ = *λ*_3_ = 1, where *ε* > 0 is the engineering strain. The long-term strain in our experiment is *λ*_10_ = 1 − *ε*_0_, with *ε*_0_ ≈ 0.42.

Under such strains, the engineering stress in the direction of compression can be written as:

3 $${P}_{HYP}({\lambda }_{1},\{{\mu }_{k},{\alpha }_{k},{\beta }_{k}\})=\mathop{\sum }\limits_{k=1}^{N}\,\frac{2{\mu }_{k}}{{\alpha }_{k}{\lambda }_{1}}({\lambda }_{1}^{{\alpha }_{k}}-{\lambda }_{1}^{-{\alpha }_{k}{\beta }_{k}}),$$

where *λ*_1_ = 1 − *ε*, with *ε* being the compressive engineering strain.

To determine the optimized parameters, we follow the standard procedure of minimizing an objective function, which we chose to be the sum-square-deviation of the experimentally measured stress from that computed by the theoretical model (Eq. (3)). However, optimizing the large number of parameters in Eq. (3) is challenging, mainly because of the presence of multiple nearly degenerate minima for our system. In order to obtain a robust model where the parameters evolve smoothly as a function of time and temperature, we first needed to avoid overfitting, and isolate a set of minimal, yet sufficient set of parameters to model the experimental data. This was done in a step-by-step procedure, as described below.

First, we found that the experimental stress-strain data for the unaged material can be fitted well using two Ogden series (i.e., *N* = 2) with a total of 6 parameters. When we optimized all six parameters to fit the observed stress-strain response under uniaxial compression, we found that the two *β* parameters are nearly zero, which is consistent with our findings that under compression without any transverse confinement there is almost no lateral bulging for compression levels of ~42% (which, for a foam of ~47% porosity amounts to roughly 90% of lock-up strain). Thus, effectively we have just four parameters to describe the unaged foam, and there is no significant difference in stress-strain response between uniaxial compression and compression under no lateral confinement.

When the foam is subjected to aging under the long-term compressive strain *ε*_0_ (with the stretch ratio being *λ*_10_ = 1 − *ε*_0_) a second network forms that is at equilibrium in the *ε*_0_ strain environment. Following Tobolosky^18^, who originally modeled this effect for rubber materials, we assume the net effect of the two networks to be additive, with the net engineering stress being:

4 $${P}_{two-network}({\lambda }_{1})={P}_{HYP}({\lambda }_{1},\{{\mu }_{k0},{\alpha }_{k0}\})+{P}_{HYP}({\lambda }_{1}/{\lambda }_{10},\{{\mu }_{k1},{\alpha }_{k1}\}).$$

In Eq. (4) we introduced a second subscript, which is 0 for the original network and 1 for the second (i.e., induced) network.

**Figure 2:**
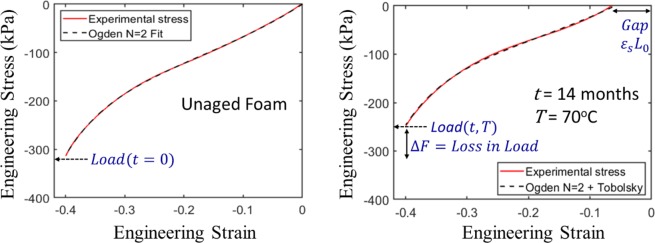
Optimized fit of Ogden + Tobolsky model to experimental stress-strain data. We use the convention of negative strain for compression. (left) unaged foam; (right) foam aged at 70 °C for 14 months. The aged foam display both the formation of a gap, which is related to the compression set (*CS*), and a loss in load, which is related to load retention (*LR*). The optimized parameters for the unaged foam were: *μ*_10_ = 383.1 kPa, *α*_1_ = 6.7, *μ*_20_ = 9.1 kPa, *α*_2_ = −6.6. The optimized parameters for the aged foam were *μ*_10_ = 246.3 kPa and *μ*_11_ = 7.4 kPa, with the other parameters being the same as that of the unaged foam (see text for model details).

To devise a model with minimal parameters we assumed a *N* = 1 Ogden model for the induced network (with *β* = 0). We also found that the aged stress-strain data can be fitted with high accuracy if the *α* exponent of the induced network, i.e., *α*_11_ is set equal to *α*_10_ (see Fig. 2 (right)). Without such constraints the model exhibits shallow local minima in the parameter space, e.g., if *α*_11_ is treated as an independent parameter, the stress-strain curve in Fig. 2(right) can be fitted only marginally better by very different sets of parameter values, which is indicative of overfitting, and which does not result in parameter sets as smooth functions of time and temperature.

Thus, altogether we have five parameters in the model, i.e., *μ*_10_, *α*_10_, *μ*_20_, *α*_20_, *μ*_11_, where the first subscript indicates the Ogden term (i.e., *k *= 1 or 2) in Eq. (1), while the second subscript indicates original (subscript 0) or induced (subscript 1) network. With such parameter choice, Eq. (4) can be explicitly written as:

5 $${P}_{two-network}({\lambda }_{1})=2{\mu }_{10}({\lambda }_{1}^{{\alpha }_{10}}-1)/({\alpha }_{10}{\lambda }_{1})+2{\mu }_{20}({\lambda }_{1}^{{\alpha }_{20}}-1)/({\alpha }_{20}{\lambda }_{1})+2{\mu }_{11}\{{({\lambda }_{1}/{\lambda }_{10})}^{{\alpha }_{10}}-1\}/({\alpha }_{10}{\lambda }_{1}),$$

where *λ*_1_ = 1 − *ε* and *λ*_10_ = 1 − *ε*_0_, with *ε*, *ε*_0_ being the (variable) engineering strain and the long-term (aging) engineering strain, respectively.

Let *L*_0_ be the thickness of the unaged foam. Then, the equilibrium thickness of the aged material is obtained as (1 − *ε*_*s*_)*L*_0_, where *ε*_*s*_ is obtained by solving the equation:

6 $${P}_{two-network}(1-{\varepsilon }_{s})=0.$$

We define compression set relative to the compression level at *t* = 0, i.e.,

7 $$CS={\varepsilon }_{s}/{\varepsilon }_{0},$$

while load retention is obtained as the ratio:

8 $$LR={P}_{two-network}({\lambda }_{10})/{P}_{HYP}({\lambda }_{10},{\mu }_{10},{\alpha }_{10},{\mu }_{20},{\alpha }_{20}).$$

When we fitted Eq. (5) to the stress-strain data at various times and temperatures, we found that the exponents *α*_10_, *α*_20_ and the shear modulus *μ*_20_ varied much less relative to the shear moduli *μ*_10_ and *μ*_11_. Thus, to simplify the aging model, we considered only parameters *μ*_10_ and *μ*_11_ to be time-dependent, and the remaining three parameters, i.e., *α*_10_, *α*_20_, *μ*_20_ were fixed at their *t* = 0 values. Having only two time-dependent parameters leads to easier interpretation of results, and because of a multitude of shallow local minima in the multi-dimensional parameter space, the above imposed constraints do not incur any significant cost in model accuracy.

Figure 2 displays the best fit of the above model to the experimental stress-strain curve of (a) the unaged foam and (b) the most extremely aged foam in this study, i.e., *t* = 14 Months at *T* = 70 °C. In both cases the model is in excellent agreement with the measured stress-strain curve. Through similar quality fitting to the stress-strain curve at various times and temperatures we can determine the aging parameters *μ*_10_ and *μ*_11_ as a function of time and temperature. Once the moduli *μ*_10_ and *μ*_11_ are determined at each time and temperature, we use Eqs (6, 7, 8) to back out the *CS* and *LR*.

**Figure 3:**
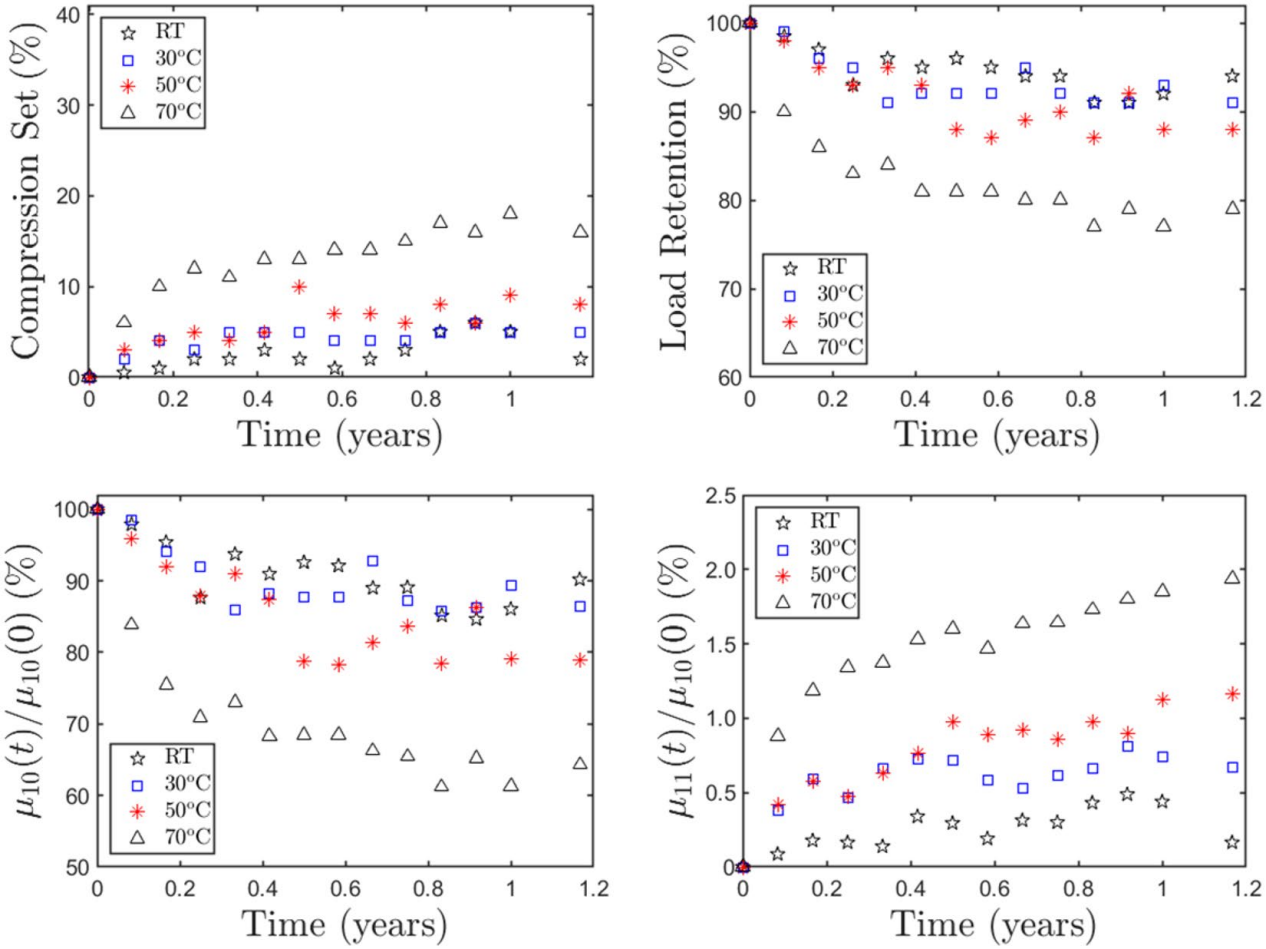
Time and temperature dependence of: compression set (*CS*); load retention (*LR*); Shear modulus *μ*_10_ of the original network; and shear modulus *μ*_11_ of the induced network. Each point in these graphs are obtained by optimized fitting of the experimental stress-strain response with the Ogden + Tobolsky model at each time and temperature, averaged over two samples for each isotherm.

Altogether this study involved a total of 8 samples aged for 14 months each at four different temperatures, and stress-strain measurements performed (roughly) once a month. Figure 3 displays the resulting *CS*, *LR*, *μ*_10_, and *μ*_11_ as a function of time and temperature, averaged over two samples per isotherm. We note here that since the present study uses different samples for different isotherms, there is sample to sample variation both within and between the isotherms. However, the noise in the resulting data (Fig. 3), especially at lower temperatures is likely more due to not achieving complete equilibrium because of slow system relaxation (i.e., long relaxation times) rather than sample differences in equilibrium properties. In spite of this limitation, there are clear trends in the plots, which can be used to build an aging model with long-term predictions.

## Time-Temperature Superposition and Long-Term Prediction

Time-temperature superposition (TTS) operates on the principle that the evolution of the property of interest at an elevated temperature over a given time-period is equivalent to the evolution of the same property over a longer time at a lower temperature^19^. Mathematically, the principle can be described by the equation:

9 $$f(t,T)=f({a}_{T}t,{T}_{ref}),$$

where *T*_*ref*_ is a reference temperature, and the factor *a*_*T*_, called the TTS shift factor^20^ is usually greater than 1 for all *T* > *T*_*ref*_. If one shifts the isotherms horizontally to appropriately scaled times (*t* → *a*_*T*_*t*), one obtains a single “master curve” at the reference temperature *T*_*ref*_). This is usually done on a logarithmic time-scale such that the procedure becomes equivalent to a rigid horizontal shift of each elevated-temperature isotherm to the right by an amount log(*a*_*T*_). In situations where the property of interest can be measured with high precision (i.e., low experimental noise), and obeys the TTS principle with great accuracy, the optimum shifts and the resulting master curve can be determined in a straightforward fashion, sometimes simply “by eye”. This is, for instance, true for the viscoelastic properties of the so-called “thermo-rheologically simple” materials^21^. However, in presence of significant noise, as is the case in Fig. 3, one needs to use objective methods of TTS shifting.

Over the years there have been a few suggested algorithms for objective TTS shifting, which include minimizing the sum of square errors in horizontal distances in the overlapping region of neighboring curves^22–26^, minimizing areas in the overlapping regions between two successive curves^27^, and minimizing the arclength of the master curve in the complex (storage, loss) modulus plane^28,29^. The last method, i.e., arclength-minimization, has recently been shown to be statistically unbiased^30^. Moreover, in case of data from only limited specimens, as is true in the present study, the work shows how to obtain more consistent shift factors through averaging over a large number of with-replacement (bootstrap) re-samples^31^.

**Figure 4:**
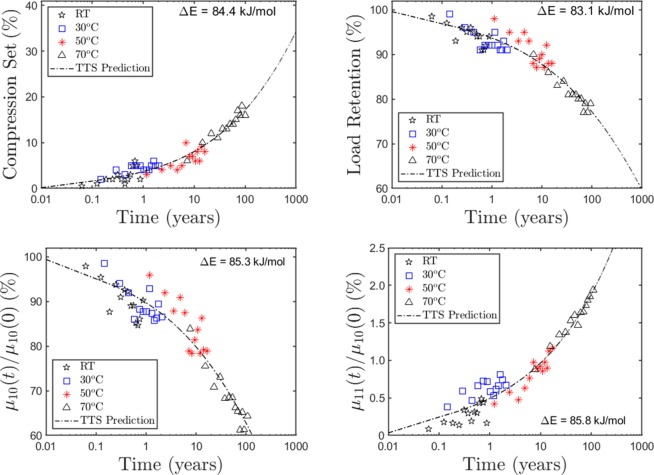
Master curves generated from the plots in Fig. 3 through time-temperature-superposition (TTS) at a reference temperature *T*_*ref*_ = 25 °C. We used the bootstrap-mean-minimum-arclength implementation of TTS as described in ref.^30^. Each property displays nearly the same Arrhenius barrier (in KJ, as indicated). Dashed lines are modified exponential fits to the master curve and can be used to make long-term predictions.

Following the method outlined in ref.^30^, the bootstrap-mean-minimum-arc algorithm was used to carry out TTS shifting of the Fig. 3 isotherms. Figure 4 displays the resulting master curves (at a reference temperature of *T*_*ref*_ = 25 °C) along with the corresponding Arrhenius activation barriers. For each property, the simulations averaged over 2000 bootstrap resamples. The dashed curves are modified exponential fits through the TTS-shifted data points and can be used as long-term prediction curves at *T*_*ref*_. Within numerical noise we observe roughly the same apparent activation barrier (Δ*E* ~ 83–86 kJ/mol) for the evolution of all four properties, which is consistent with a common dominant mechanism at the molecular/network level governing the evolution of these properties.

**Figure 5:**
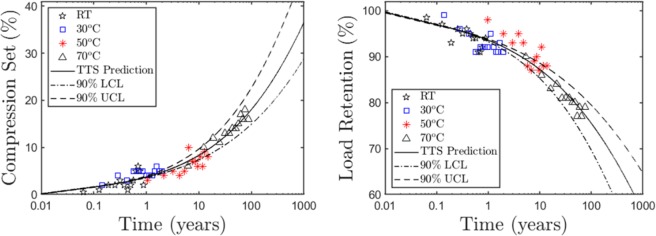
90% confidence margins in the long-term prediction of compression set and load retention, using a second-order bootstrap method, as described in ref.^30^. The shift factors are constrained by the Arrhenius model (Eq. (10) in text). The resulting margins are more influenced by latter-time measurement noise at elevated temperatures.

Finally, as noted in ref.^30^, the bootstrap-mean-minimum-arc method yields a sampling distribution of activation barriers that is normal with a near-zero-bias sampling mean. This allows us to construct confidence interval (i.e., margin curves) for the long-term prediction. Figure 5 displays the 90% CI margins for *CS* and *LR*. We interpret these margins as the envelop curves within which the true *CS* (or *LR*) evolution curve (at *T*_*ref*_ = 25 °C) is expected to lie with a probability of 90%. We observe that lower-temperature measurement noise at short times do not influence the margins as much as the noise in latter-time measurements, especially at elevated temperatures.

It should be noted here that the margin curves in Fig. 5 are constructed based on the Arrhenius model, i.e., under the assumption that the shift factors at different temperatures are not independent variables, but rather follow the constraint^30^:

10 $$\log ({a}_{T})={E}_{a}\{\frac{1}{{k}_{B}{T}_{ref}}-\frac{1}{{k}_{B}T}\},$$

where *E*_*a*_ is the activation barrier, *T*_*ref*_ the reference temperature, and *k*_*B*_ the Boltzmann constant. This imposes strong restrictions on the master curve prediction (at *T* = *T*_*ref*_) for shorter times, and thus even moderate noise in low-temperature data points can drive them outside the margins. However, given that the goal of TTS is long-term prediction, this does not impose any practical limitations to the method. In fact, imposing a constraint like an Arrhenius model adds stability to the long-term master curve, and puts the margin curve calculation on a firm statistical footing. As mentioned in the previous section, the noise in lower temperature data likely arises out of slow relaxation, which results in the system perhaps not completely reaching equilibrium at the time of measurements. The Arrhenius-based smooth margins are (rightly) not influenced by such noise.

## Discussion and Summary

In this paper we described the development of a thermal-age-aware constitutive model for a 3D printed foam under long-term compressive strains. It is based on the Ogden hyperfoam model for stress-strain response, originally developed for stochastic cellular materials. The effect of thermal aging is modeled through the creation of a second network that is in equilibrium under the strained condition (i.e., compression in our case). Borrowing from Tobolsky’s work on rubber, the net effect of the two networks is assumed to be additive. The first (original) network is modeled by *N* = 2 Ogden, while the second (induced) network is modeled by *N* = 1 Ogden. For transversely unconfined compression experiments we find insignificant lateral bulging for strains up to 90% of lock-up, which translates into near-zero *β* parameters (i.e., Poisson’s ratio ≈ 0). Thus, our model has a total of five parameters, out of which only two shear moduli, i.e., *μ*_10_ and *μ*_11_ are assumed to be age-dependent (i.e., functions of time and temperature). Through accurate fitting of experimentally measured stress-strain curves at different times and temperatures, the model yields the functions *μ*_10_(*t*, *T*) and *μ*_11_(*t*, *T*). Additionally, we also back out quantities like compression set (*CS*) and load retention (*LR*) out of the model (see Eqs 5 through 8), two fundamental metrics that are used to quantify the mechanical/structural aging of polymeric components in real systems. The method of Time-temperature-superposition (TTS) is then used to create long-term prediction curves of each of these four quantities, along with margin curves for uncertainty quantification. We expect the same model to describe thermal aging of stochastic foams as well, although we need experimental aging data for obtaining optimized parameters for such systems.

For the printed foam, we observe roughly the same activation barrier (Δ*E* ~ 83–86 kJ/mol) for the evolution of all four properties (i.e., *CS*, *LR*, *μ*_10_, and *μ*_11_), which is consistent with a single dominant molecular/network level mechanism governing the aging of all such properties. However, to associate the obtained activation barrier with specific structure-relaxation or damage-formation modes in a complex macromolecular system such as a filled polymeric network is non-trivial, and warrants further analysis, e.g., analyzing microstructural changes in X-ray computer-tomography images^32–34^, attempting to assess changes in molar mass distributions and chain entanglements through methods such as mechanical^35^, dielectric spectroscopy^36^, solid state NMR^37^ and solvent-swelling experiments^38,39^, as well as realistic multiscale simulations^40^.
